# Antenatal Care Utilisation and Content between Low-Risk and High-Risk Pregnant Women

**DOI:** 10.1371/journal.pone.0152167

**Published:** 2016-03-24

**Authors:** Ping Ling Yeoh, Klaus Hornetz, Maznah Dahlui

**Affiliations:** 1 Department of Social and Preventive Medicine, Faculty of Medicine, University of Malaya, Kuala Lumpur, Malaysia; 2 German Development Cooperation, Berlin, Germany; 3 Mediconsult Sdn. Bhd., Ampang, Malaysia; National Institute of Health, ITALY

## Abstract

**Background:**

The purpose of antenatal care is to monitor and improve the wellbeing of the mother and foetus. The World Health Organization recommends risk-oriented strategy that includes: (i) routine care to all women, (ii) additional care for women with moderately severe diseases and complications, (iii) specialised obstetrical and neonatal care for women with severe diseases and complications. Antenatal care is concerned with adequate care in order to be effective. Measurement for adequacy of antenatal care often applies indexes that assess initiation of care and number of visits. In addition, adequacy of care content should also be assessed. Results of studies in developed settings demonstrate that women without risk factors use antenatal services more frequently than recommended. Such over-utilisation is problematic for low-resourced settings. Moreover, studies show that a substantial proportion of high-risk women had utilisation or content of care below the recommended standard. Yet studies in developing countries have seldom included a comparison between low-risk and high-risk women. The purpose of the study was therefore to assess adequacy of care and pregnancy outcomes for the different risk groups.

**Methods:**

A retrospective study using a multistage sampling technique, at public-funded primary health care clinics was conducted. Antenatal utilisation level was assessed using a modified Adequacy of Prenatal Care Utilisation index that measures the timing for initiation of care and observed-to-expected visits ratio. Adequacy of antenatal care content assessed compliance to routine care based on the local guidelines.

**Results:**

Intensive or “adequate-plus” antenatal care utilisation as defined by the modified index was noted in over half of the low-risk women. On the other hand, there were 26% of the high-risk women without the expected intensive utilisation. Primary- or non-educated high-risk women were less likely to have a higher antenatal care utilisation level compared with tertiary educated ones (OR = 0.20, P = 0.003). Half of all women had <80% of the recommended antenatal care content. A higher proportion of high-risk than low-risk women scored <80% of the routine care content (p<0.015). The majority of the additional laboratory tests were performed on high-risk women. Provision of antenatal education showed comparatively poor compliance to guidelines, more than half of the antenatal advice topics assessed were rarely provided to the women. High-risk women were associated with a higher prevalence of adverse pregnancy outcome.

**Conclusions:**

Disproportionate utilisation of antenatal care according to risk level of pregnancy indicates the need for better scheduling of care. The risk-oriented approach often results in a tendency to focus on the risk conditions of the women. Training interventions are recommended to improve communication and to help healthcare professionals understand the priorities of the women. Further studies are required to assess the reason for disproportionate utilisation of antenatal care according to risk level and how delivery of antenatal advice can be improved, reviewing both user and provider perspectives.

## Introduction

In an environment of rapid economic development and associated positive changes in demographic development, poverty reduction and lifestyle, Malaysia has achieved remarkable progress in healthcare and health status [1]. Despite excellent maternal-child-health services coverage [2, 3] in the past decades, progress in pregnancy outcomes is stagnating. The development of stillbirth and perinatal mortality over time show a slight upward trend in the past decade [4, 5]. Maternal mortality ratio has been stagnant at around 28–30 per 100,000 live-birth since many years [6, 7]. Studies on underlying issues and options for change is sparse and lacked data on content of care.

The purpose of antenatal care (ANC) is to monitor and improve the wellbeing of the mother and foetus, detect complications, respond to women’s complaints, prepare for birth, and promote healthy behaviours [8]. In the 90’s, randomised trials have been conducted to compare the standard model of ANC with a new model of care [9]. The new model applied a more cost-efficient risk-oriented approach emphasising on actions known to be effective in improving maternal or neonatal outcomes [9]. Promoting the latter strategy, the World Health Organization (WHO) recommends a minimum of four ANC visits for uncomplicated pregnancy. The risk-oriented ANC strategy involves: (i) routine care to all women, (ii) additional care for women with moderately severe diseases and complications, and (iii) specialised obstetrical and neonatal care for women with severe diseases and complications [8]. Malaysia adopts a similar risk-oriented approach, referring to the British model of care [10].

ANC is concerned with adequate care in order to be effective. Guidelines have been developed to provide guidance on adequate initiation of care, number of visits and content of routine care [11–13]. Measurement for adequacy of ANC often applies indexes that assess initiation of care and number of visits [14, 15]. For example, Kotelchuck’s Adequacy of Prenatal Care Utilisation (APNCU) index [16] which is considered the standard and most used index for ANC utilisation [14, 17]. In addition to measuring the initiation and number of visits, adequacy of content of care should be assessed [8, 18].

Various recent studies have assessed ANC utilisation and/or content in developing countries [19–24]. However, these studies have seldom included a comparison between low-risk and high-risk women. Comparison of ANC utilisation and content by risk level in developed settings [25–29] has been more frequently studied. Results of these studies demonstrate that women without risk factors use ANC services more frequently than recommended [26, 28, 30]. Such over-utilisation is problematic for low-resourced settings. At the same time, these studies show that for a substantial proportion of high-risk women, the utilisation of ANC [26, 28, 29] or the content of care delivered [25] is below the recommended standard.

This study was part of a larger research focusing on adequacy of ANC, associated factors and pregnancy outcomes in Malaysia. It aimed to contribute towards identifying factors leading to stagnating pregnancy outcomes in Malaysia, and to inform strategy formulation towards change. The purpose of this particular paper was to assess adequacy of care and pregnancy outcomes for the different risk groups.

### Provision and organisation of antenatal care in Malaysia

Malaysia has achieved Universal Health Coverage in the 1980s. Health services are delivered through a network of tax-funded public healthcare organisations and private providers. Except for a registration charge of USD 0.30 per visit, ANC services provided by public sector primary health clinics are free-of-charge [1].

The Ministry of Health’s (MOH) ANC schedules referred to the British guidelines [11], which recommend ten and seven visits, respectively, for primigravida and multigravida with uncomplicated pregnancy. Two routine visits to a medical doctor are included for each uncomplicated pregnancy attending ANC at the public sector health clinics [10]. The clinics use a risk assessment system to assess and classify women according to a listing of risk factors (S1 Table) at several gestational periods. The result of the risk assessment classifies the women into one of four colour codes that function as managerial tools to determine care providers, locations of ANC and delivery [10].

The public sector primary health clinics in Malaysia adopted a dual-record system whereby the women carry their own case notes and the clinics keep a duplicate set.

## Methods

In this paper, adequacy of ANC measures two dimensions. One, adequacy of utilisation assessed gestational age at initiation of care and observed-to-expected visits ratio which was summarised as one combined index [16]. Two, adequacy of content that assessed compliance to recommended routine care according to the ANC guidelines of Malaysia [10].

### Study design and population

A retrospective cohort study was conducted to assess the utilisation and content of ANC among women attended public-funded health clinics in Selangor, Malaysia. The study design and population have been described elsewhere [31]; a multistage sampling procedure was applied in the selection of the health clinics and pregnant women. Six out of 58 health clinics in the state were selected. Patient records/information were anonymized and de-identified prior to analysis. The study was approved by the Research and Ethics Committee of University Malaya, Malaysia and the Medical Research and Ethics Committee of Ministry of Health, Malaysia.

### Description of variables

#### Risk level of pregnancy

Risk level for each woman was assessed according to the MOH’s risk assessment system outlined in S1 Table which classified the women into one of four colour codes [10]:

- **White**—Pregnancy without risk factor, follow-up by nursing staff at clinic.
- **Green**—Pregnancy with low-risk factor, refer to medical officer at clinic for decision on subsequent provider (follow-up by medical officer or nursing staff at health clinic).
- **Yellow**—Pregnancy with high-risk factor, refer to the Obstetrics and Gynaecology specialist at hospital or Family Medicine Specialist at clinic within 48 hours.
- **Red**—Pregnancy with extreme high-risk factor, seek urgent medical attention and refer to hospital immediately.

For the analysis, low-risk denotes pregnancies without risk or with only low-risk factors (white and green tags), and high-risk refers to pregnancies with high-risk factors (yellow and red tags).

#### Adequacy of antenatal care utilisation

ANC utilisation assessed gestational age at initiation of care and observed-to-expected visit ratio based on the Adequacy of Prenatal Care Utilisation Index (APNCU index) [16]. This index includes two separate indices for adequacy that are combined into a single summary index. The first considered the gestational age at initiation of ANC. The second compared the actual number of visits between initiation of ANC and delivery to the expected number of visits based on the American College of Obstetricians and Gynaecologists (ACOG) recommended visits. These two indices—gestational age at initiation and observed-to-expected visit ratio—were combined into a single summary antenatal care utilisation index [16]. According to the original APNCU index, the cut-off points for the observed-to-expected visits ratio is categorised into: Adequate-plus (≥ 110% of expected visits), Adequate (80%-109%), Intermediate (50%-79%), and Inadequate (<50%).

The original APNCU index was based on 13 visits as recommended by ACOG for a 40-week pregnancy [13]. The Malaysian guidelines recommend ten and seven visits for primigravida and multigravida, respectively [10], lower than the number of visits used by the original APNCU. When the cut-off points of the original APNCU were applied to the Malaysian guidelines with lower recommend visits, one additional observed visit compared to expected visits would fall into the ≥ 110% (adequate-plus) range, presenting a bias [31]. This study therefore modified the index by raising the cut-off points of the observed-to-expected visit ratio to accommodate the lower recommended visits of the local guidelines. The modified cut-off points of the ratio became: ≥ 130% (adequate-plus), 90–129% (adequate), 60–89% (intermediate), and <59% (inadequate). For analysis of the results, ANC utilisation was grouped into three categories: “adequate-plus” (intensive utilisation with ≥ 30% higher visits than recommended), “adequate”, and “inadequate” (intermediate was grouped under inadequate).

There are limitations to the APNCU Index. It is a measure for adequacy of ANC utilisation which does not measure the adequacy of ANC content. It also does not adjust for risk conditions of pregnant woman because the recommended number of visits of the ACOG is for women with uncomplicated pregnancies [16]. To address these limitations, the present study incorporated an assessment on adequacy of ANC content. It also included the independent variable for risk level of pregnancy to enable analysis of the women by their risk level. The risk-oriented approach established that high-risk women would have additional visits due to the need to monitor their risk conditions. They were therefore expected to have intensive utilisation that fall under the “adequate-plus” category.

#### Adequacy of antenatal care content

As described elsewhere [31], adequacy of content assessed compliance to recommended routine ANC based on the MOH guidelines [10, 32]. The assessment components consisted of physical examination, health screening, case management and antenatal education. ANC content scores were tabulated using the compliance criteria for scoring (S2 Table). ANC content was categorised as inadequate (<80% compliance score) or adequate (≥ 80% compliance score).

#### Additional tests

Assessment of additional testing included selected tests that were not part of the routine ANC and were performed according to the risk conditions of the women. These included Modified Glucose Tolerance Test (MGTT), urine Full Examination/Microscopic Examination (FEME), blood sugar profile (BSP), haemoglobin A1c or glycated haemoglobin test for diabetes (HbA1c), full blood picture (FBP), iron studies and haemoglobin electrophoresis.

#### Pregnancy outcomes

The main pregnancy outcomes defined and analysed were adverse foetal outcomes and maternal complications. Adverse foetal outcomes included preterm birth (<37 weeks gestation at birth), low birth weight (LBW, <2,500 g at birth), and stillbirth (intrauterine death at ≥ 22 weeks gestation or over 500 g). Maternal complications in this study included women with one of these conditions: retained placenta, postpartum haemorrhage, impending eclampsia/ preeclampsia, postnatal high blood pressure, postnatal infection (infected wound or systemic, e.g., fever), postnatal severe anaemia, unknown reason for admission or long hospital stay, maternal death (there was only one maternal death that met the eligibility criteria).

### Data analysis

Cross-tabulation was performed to compare the level of utilisation and content adequacy among low-risk and high-risk women. Descriptive statistics were used to describe the documented antenatal advice/interventions among these two groups. To explore further the factors associated with ANC utilisation level among the low and high-risk women, the analysis used multivariable ordinal regression to examine the two groups separately. The link function of “complementary log-log” was chosen because the highest category is more probable. A full model containing all of the variables identified—maternal age, ethnicity, maternal education, maternal occupation, parity, risk level and type of clinics—was first constructed for all women. A stepwise backward selection method was then employed using only the significant variables (P<0.05) from the previous full model analysis. A split file function was employed to analyse the low and high-risk women separately. Cross-tabulation was used to examine the pregnancy outcomes by women's risk level.

## Results

### Respondents characteristics

A total of 522 eligible women’s record was analysed. Out of which, 72% were categorised as low-risk, and 28% were high-risk. The mean maternal age at the first visit was 28.3 and 29.6, respectively, for low-risk and high-risk women. The majority of ANC attenders were from the age group 20- to 34-years-old, representing 87% of the low-risk women and 76% of the high-risk women. Women aged 35 and above constituted 11% and 20% of the low- and high-risk groups, respectively. There were a total of 11 teenage pregnancies.

Most of the women were ethnic Malay, 79% and 67%, respectively for the low- and high-risk groups. The official ethnic composition of Selangor showed that both Malay and indigenous people formed 57% of the total population, along with Chinese (29%), Indians (14%) and others (0.8%) [33]. This inferred Malays used the ANC services of the public-funded clinics in higher proportions than other ethnicities, consistent with the finding of national survey that showed a higher proportion of Malays used public facilities [34].

Over half of the women had secondary education. A higher proportion of the low-risk than high-risk women had tertiary education (40% vs 31%). As for the women’s occupations, it appears that a higher proportion of low-risk women held managerial or professional posts than high-risk. There was a lower proportion of the low-risk group that had no formal employment. The mean parity was 1.1 and 1.4, respectively, for low and high-risk. The distribution of nulliparity and multiparity was similar between the two groups (S3 Table).

### Adequacy of antenatal care utilisation

In total, there was a large proportion of women (63%, 330/522) with “adequate-plus” or intensive ANC utilisation, while 21% (107/522) of the women had “inadequate” utilisation. Table 1 shows that low-risk women had a significantly larger proportion in the “adequate” utilisation category (93%) compared to other categories of utilisation (inadequate 70%, adequate-plus 67%). In comparison, high-risk women had a significantly lower proportion in the “adequate” utilisation category (7%), but a higher proportion in the “adequate-plus” (33%) and “inadequate” (30%) categories.

**Table 1:** Antenatal care utilisation level among low- and high-risk women in public-sector health clinics of Selangor state (n = 522).

Risk level	Sample size, n (%)	Antenatal care utilisation level, n (%)			p
		Inadequate	Adequate	Adequate-plus	
Low-risk	375 (71.8)	75 (70.1)	79 (92.9)	221 (67.0)	<0.001
High-risk	147 (28.2)	32 (29.9)	6 (7.1)	109 (33.0)	
Total	522 (100.0)	107 (100.0)	85 (100.0)	330 (100.0)	

Twenty-six percent (38/147) of the high-risk women had either inadequate (32/147) or adequate utilisation (6/147). Considering that high-risk women require more frequent visits to monitor their risk conditions, they were expected to have “adequate-plus” utilisation. These 38 high-risk women therefore were deemed having inappropriate low utilisation. Out of these 38 high-risk women, five were ever referred to a hospital for additional consultation before 28 weeks, 16 were ever referred to a hospital at 28 weeks onwards, and 17 had no documented referral to a hospital.

When the gestational age at initiation and observed-to-expected visit ratio indices where analysed separately, 82% of the women had adequate and 18% had inadequate initiation of care according to the modified APNCU index (S3 Table). The distribution between the two groups was almost similar: 82% and 18%, respectively for adequate and inadequate among the low-risk compared to 80% and 20%, respectively among the high-risk.

As for adequacy of observed-to-expected visits ratio, 95% of the women had adequate and 5% had inadequate recommended visits. The distribution is the same between the two groups.

#### Factors associated with antenatal care utilisation

When examining the factors associated with ANC utilisation level, multivariate regression for all of the women showed that maternal age, maternal education, parity, risk level and type of clinic made statistically significant contributions to the analysis model. Ethnicity and maternal occupation were found to have no significant influence on adequacy of ANC utilisation level (p>0.05, Table 2). When these significant variables were examined among low- and high-risk women separately, it was found that maternal age, parity and clinic type had statistically significant associations with utilisation level among the low-risk group (p>0.05). Maternal education was the only factor that made a statistically significant contribution to utilisation level among the high-risk group (p>0.05).

**Table 2:** Results of ordinal regression on factors associated with high antenatal care utilisation among low- and high-risk women in public-sector health clinics of Selangor state (n = 522).

	All women		*Low-risk		*High-risk	
Characteristics	OR (95% CI)	P	OR (95% CI)	P	OR (95% CI)	P
**Socio-demographic**						
Age group:						
20–34	1.80 (1.22–2.66)	0.003	2.03 (1.28–3.20)	0.002	1.27 (0.60–2.72)	0.534
< = 19 & 35+	1.00		1.00		1.00	
Ethnicity:						
Malay	1.62 (0.70–3.78)	0.262				
Chinese	0.85 (0.34–2.11)	0.726				
Indian	1.15 (0.45–2.93)	0.770				
Indigenous people	1.00					
Education level:						
Primary or no education	0.44 (0.22–0.91)	0.027	0.69 (0.28–1.68)	0.415	0.20 (0.07–0.58)	0.003
Secondary	0.82 (0.53–1.27)	0.367	0.92 (0.65–1.30)	0.638	0.64 (0.28–1.44)	0.280
Tertiary	1.00		1.00		1.00	
**Socio-economic Status**						
Occupation (PW):						
Managers, professionals and associate professionals	0.87 (0.54–1.41)	0.571				
Non managerial and nonprofessional workers (incl. clerical support, service/sales, craft and related trades, plant/machine, elementary workers)	1.01 (0.71–1.43)	0.956				
Non formal employment (housewives, students, unemployed)	1.00					
Obstetric/ risk factor						
Parity:						
Multiparous	2.15 (1.57–2.96)	<0.001	2.58 (1.82–3.70)	<0.001	1.11 (0.55–2.26)	0.767
Nulliparous	1.00		1.00		1.00	
Risk level of pregnancy:						
Low-risk	0.51 (0.35–0.73)	<0.001	NA		NA	
High-risk	1.00					
**Provider**						
Clinic type						
<150	1.00		1.00		1.00	
150–300	0.73 (0.47–1.15)	0.173	0.74 (0.45–1.20)	0.219	0.59 (0.21–1.63)	0.306
301–500	0.53 (0.34–0.83)	0.006	0.52 (0.32–0.86)	0.010	0.53 (0.19–1.48)	0.224

* stepwise backward selection model using significant variables (P<0.05) from the full model for “all women”.

Among the low-risk women, this study found that those aged 20–34 years were more likely to have higher ANC utilisation than women aged ≤ 19 or ≥ 35 years. The odds of women 20–34 years of age having higher ANC utilisation were two times that of women aged ≤ 19 or ≥ 35 years (OR = 2.03, 95%CI = 1.28–3.20, P = 0.002).

Multiparous women were more likely to have higher ANC utilisation than nulliparous women in the low-risk group. The odds for multiparous women having a higher ANC utilisation level were over twice the odds for nulliparous women (OR = 2.58, 95%CI = 1.82–3.70, P<0.001).

Among the low-risk women, higher ANC utilisation was observed among those who visited the smaller clinics (reference OR = 1.00) compared to the biggest clinics (OR = 0.52, 95%CI = 0.32–0.86, P = 0.010).). The odds of having higher ANC utilisation among those who attended the smallest clinics (<150 expected daily patients load) were twice those among the biggest clinics (301–500 expected daily patients). There were no significant differences between those attending clinics with 150–300 expected daily patients and the smallest clinics (P = 0.173) or the biggest clinics (overlapping 95% CI of the coefficients).

Maternal education was the only factor significantly associated with utilisation level among the high-risk women. Primary or non-educated high-risk women were less likely to have higher ANC utilisation than those with tertiary education (OR = 0.20, 95%CI = 0.07–0.58, P = 0.003). The odds of tertiary educated women having higher ANC utilisation level were five times the odds for primary educated women. There was no significant difference between secondary and tertiary education (P = 0.280), nor between primary and secondary education.

### Adequacy of antenatal care content

#### Compliance to routine antenatal care content

The mean content score among the low-risk women was significantly higher than that among the high-risk women statistically, although it was a small difference at 78% and 76%, respectively. Of all the women, 52% had <80% of the routine ANC content documented. As presented in Table 3, low-risk women had a larger proportion in the adequate content category (77%) compared to inadequate (67%). In contrast, among the high-risk women, 23% had adequate content and 33% had inadequate content.

**Table 3:** Antenatal care content adequacy level among low- and high-risk women in public-sector health clinics of Selangor state (n = 522).

Risk level	Sample size, n (%)	Antenatal care content adequacy, n (%)		
		Inadequate (<80%)	Adequate (≥80%)	p
Low-risk	375 (71.8)	181 (67.0)	194 (77.0)	0.015
High-risk	147 (28.2)	89 (33.0)	58 (23.0)	
Total	522 (100.0)	270 (100.0)	252 (100.0)	

With regards to different antenatal advice topics, some of the advice was more universally provided, e.g., antenatal dietary advice, whereas advice on physical exercise and postnatal care was seldom given (Table 4). Majority of the advice was less frequently provided to the high-risk women than the low-risk. This included family planning (54% vs 72%), preparation for birth (56% vs 81%), and birth process (75% vs 88%). In total, 26% of the women received advice on common disorders in pregnancy, which was more often provided to high-risk than low-risk women (40% vs 21%).

**Table 4:** Antenatal advice provided and abdominal ultrasound performed among low- and high-risk women in public-sector health clinics of Selangor state (n = 522).

	All women (n = 522)	Low-risk (n = 375)	High-risk (n = 147)
**Antenatal advice topics based on official checklist [32]:**	**Given, %**	**Given, %**	**Given, %**
nutritional/dietary advice—antenatal	99.2	98.9	100.0
nutritional/dietary advice—postnatal/ breastfeeding	1.3	1.3	1.4
recommendations for family planning/ contraception	66.7	71.7	53.7
preparation for birth	73.9	81.1	55.8
birth process (signs/symptoms and related advice)	84.3	88.0	74.8
common discomfort during pregnancy and solutions	22.6	23.5	20.4
recommendations for breastfeeding	71.3	72.0	69.4
common disorders in pregnancy (at least 2 topics–pregnancy induced hypertension, preeclampsia/ impending eclampsia, gestational diabetes mellitus, anaemia, bleeding during pregnancy)	26.1	20.5	40.1
early booking	6.1	6.7	4.8
foetal development	12.8	14.9	7.5
exercise antenatal/ postnatal	3.3	3.5	2.7
newborn care, baby bathing	0.4	0.3	0.7
jaundice baby care	19.7	21.6	15.0
postnatal care	5.2	5.6	4.1
**Abdominal ultrasound performed:**			
≥ 2 times	78.4	75.7	85.0
First ultrasound by 24 gestational weeks	82.2	81.3	84.4
**Abdominal ultrasound:**	**Mean**	**Mean**	**Mean**
Gestational age at first ultrasound, in weeks [excluding women who had their first ultrasound done elsewhere prior to the first visit in which the gestational age was not documented, and women without ultrasound]	18.1	18.3	17.7
total ultrasounds performed (all providers), in #	2.6	2.4	3.2

The mean gestational age for performing the first ultrasound was 18 weeks, within the range of the local guidelines to have the first ultrasound by 24 weeks. Comparison by risk level showed no significant difference in terms of initiation period between low-risk and high-risk women.

High-risk women had slightly higher average total ultrasounds (3.2 vs 2.4, Table 4). A higher proportion of high-risk than low risk women had at least two ultrasounds (85% vs 76%). A slightly higher proportion of high-risk than low-risk women had their first ultrasound by 24 weeks (84% vs 81%).

#### Additional laboratory tests

Table 5 shows that MGTT was the most frequently performed additional test, constituting 72% of the women. Eighty percent of high-risk women were ever tested compared to 69% of low-risk women. The 69% of low-risk women tested were all negative.

**Table 5:** Additional laboratory tests performed among low- and high-risk women in public-sector health clinics of Selangor state (n = 522).

	All women (n = 522)	Low-risk (n = 375)	High-risk (n = 147)
Additional tests, n (%)	Tested	Tested	Tested
MGTT	376 (72.0)	258 (68.8)	118 (80.3)
urine FEME	66 (29.9)	105 (28.0)	51 (34.7)
BSP	86 (16.5)	3 (0.8)	83 (56.5)
HbA1c	51 (9.8)	0 (0.0)	51 (34.7)
FBP	33 (6.3)	10 (2.7)	23 (15.6)
iron studies	28 (5.4)	8 (2.1)	20 (13.6)
Hb analysis (electrophoresis)	19 (3.6)	7 (1.9)	12 (8.2)

modified glucose tolerance test (MGTT), urine full examination/microscopic examination (FEME), blood sugar profile (BSP), haemoglobin A1c or glycated haemoglobin test (HbA1c), full blood picture (FBP), haemoglobin electrophoresis (Hb analysis).

Almost all blood sugar profile tests, which are used to monitor gestational diabetes mellitus, were performed on high-risk women (83 out of 86 tests). HbA1c tests were documented for 10% of the women, who were all high-risk.

Majority of the investigations for anaemia–full blood picture, iron studies, and haemoglobin electrophoresis–were performed on the high-risk group (Table 5). Several low-risk women were also tested. Thirty percent of the women had urine FEME. Thirty-five percent of high-risk women had urine FEME documented compared to 28% of low-risk women.

### Pregnancy outcomes and risk level

Table 6 shows that high-risk women were associated with higher prevalence of adverse foetal outcomes than low-risk (22% vs 14%, P = 0.022). Although maternal complications were not significantly influenced by risk level statistically (P = 0.138), it appears that high-risk women had slightly more maternal complications (9% vs 5%).

**Table 6:** Pregnancy outcomes among low- and high-risk women in public-sector health clinics of Selangor state (n = 522).

	All women (n = 521)	Low-risk (n = 374)	High-risk (n = 147)	p
	n (%)	n (%)	n (%)	
*Adverse foetal outcome (n = 86)	86 (16.5)	53 (14.2)	33 (22.4)	0.022
Maternal complications (n = 33)	33 (6.3)	20 (5.3)	13 (8.8)	0.138
Adverse pregnancy outcome (all) (n = 111)	111 (21.3)	30 (18.7)	41 (27.9)	0.021

*including preterm birth, low birth weight and stillbirth

## Discussion

### Antenatal care utilisation

The distribution of the women with intensive/ “adequate-plus” utilisation by risk level is similar to the finding of studies in the United States in which approximately 64–75% of the “adequate-plus” category (based on the original APNCU classification) were women without maternal medical risk factors [26, 28]. This illustrates the issue of high utilisation of ANC among low-risk women who were not expected to have more ANC visits than recommended. Intensive utilisation among low-risk women may indicate inappropriate use of services which will have implication on the cost of healthcare delivery and possibly the attention for high-risk women who are expected to need more visits.

There were 26% of the high-risk women that had inappropriately low utilisation given that high-risk women were expected to have intensive utilisation for frequent visits to monitor their risk conditions. Around half of these 26% of the high-risk women were referred to a hospital for additional care. However, nearly the other half of these women had no documented referral to a hospital. This could partly be explained by possible non-documentation of these referrals. Some of the women might also have, out of their own initiative, attended private clinics parallel to receiving ANC at the public clinics which was not reported to the public clinics. This implies the need for further research.

#### Factors associated with antenatal care utilisation

Studies on factors associated with ANC utilisation generally did not analysed the factors separately by the risk level of the women. Comparisons with other studies can only draw on their overall findings. There have been differing findings concerning maternal age and ANC utilisation. Studies in developed settings have reported that women <20 years old were less likely to have high utilisation than older women [26, 29], and higher utilisation in general among women ≥ 35 years has been reported [26, 28–30]. In some developing settings, increased age was found to be associated with higher usage [19, 23]. It was also found that older women, who generally had higher utilisation than younger women, received better quality ANC [19]. Some studies have reported no significant effect of maternal age on ANC utilisation [20, 21]. On the other hand, there was a study in a developing setting that reported higher utilisation among younger women because the health sector gave priority to primigravida cases, who are also generally younger, to receive ANC [24]. It was also reported that perceived risk associated with first pregnancy contributes to higher utilisation among younger women [24]. These differences indicate that the influence of maternal age on ANC utilisation differs according to the setting of a study.

In the case of the present study, women aged ≤ 19 may be less educated or less informed about the importance of ANC utilisation. As for women aged ≥ 35 without high-risk factors, these women might use ANC less frequently due to self-perceived experience from previous pregnancy. Complication-free pregnancy has been associated with low utilisation as the women might not perceive ANC visits as necessary [20, 21]. Moreover, these older women might not have time for ANC due to their responsibilities such as caring for other children [21, 23, 24].

Other studies have reported that multiparous women, especially those with high parity, tend to be associated with no and/or low utilisation of ANC because they are more likely to rely on their past pregnancy experience and might not feel the need for ANC [21, 23, 24]. The higher level of ANC utilisation reported among multiparous women in this study could be explained by the difference in the recommended ANC schedule between primigravida (also refers as nullipara) and multigravida. A primigravida was recommended to have ten ANC visits, whereas it was seven visits for a multigravida. The multiparous women’s heath seeking behaviour could be influenced by the experience of their first pregnancy; therefore, they might continue to follow a schedule similar to that of their first pregnancy. In such instances, a multigravida with ten visits would be categorised as having intensive or “adequate-plus” utilisation following the classification of the index in this study.

Among the low-risk women, higher ANC utilisation was observed among those who visited the smaller clinics compared to the biggest clinics. In the context of this study, the biggest clinics were located in the most populated districts. These urban clinics were known to have a higher actual user load than their planned capacity [1]. The resulting long wait may deter the users from using the services more than necessary.

Consistent with many studies in developing settings, maternal education was a strong predictor of ANC utilisation; higher education attainment increases the frequency of ANC utilisation in developing settings [19, 21–23]. By contrast, the proportion of inadequate utilisation increases with higher educational level in developed settings [26, 28]. However, a study in Finland showed under-utilisation of ANC was less frequent among more highly educated women [29].

In essence, the large proportion of intensive utilisation among low-risk women could be due to a combination of factors discussed earlier. Majority of the women were from the age-group of 20–34 years. Some of these women were pregnant for the first time, hence, might have higher utilisation due to perceived risk associated with first pregnancy. Nevertheless, multipara had higher utilisation than nullipara. As discussed earlier, the multipara’s health seeking behaviour were likely to be influenced by the ANC schedule of their first pregnancy. On the other hand, the finding of high-risk women without intensive utilisation was partly because they were referred to a hospital for additional care. As for the other half of these women who had no documented referral, this might be due to documentation issue or attending private clinics in parallel which was not reported to the health clinics.

### Antenatal care content

Few studies compared the extent of compliance to ANC content according to the women's risk level. In a study conducted in a developed setting, a higher proportion of high-risk women received ANC content with documented compliance of ≥ 80% compared to the low-risk women, 34% vs 24% [25]. This differs from the present study, which recorded a lower proportion of high-risk women receiving ≥ 80% of the recommended ANC content. Our observation from the review of the records learnt that the care given to high-risk women tended to focus on their high-risk condition; lesser attention was given on routine care that is supposed to be offered to all women. For example, the care for a woman with severe anaemia or gestational diabetes mellitus would heavily focus on haemoglobin or blood sugar monitoring, with lesser attention on other care. This is consistent with studies which show healthcare professional are more inclined to view medical risk in isolation; ANC for high-risk women tend to focus on their high-risk factors [35–38]. However, what women with high-risk pregnancies want from their healthcare professionals may not concur with what healthcare professionals think is important [35]. Healthcare professional needs to understand the importance of providing routine care to all women as it is designed to monitor and improve the wellbeing of the mother and foetus, detect other complications, respond to the women’s complaints, prepare for birth, and promote healthy behaviours [8].

Regarding antenatal advice, it appeared that the nurses attached different levels of importance to different antenatal advice topics [31]. Some of the advice was more universally provided than others, for example, antenatal dietary advice, signs and symptoms of birth. In addition, the observation validated the orientation towards risk management in the care of high-risk women, majority of the general/ non-risk focused advice was less frequently provided to the high-risk women. The healthcare professional prioritised the need for information according to the risk-factors of the women and overlooked other needs. This finding could be explained by the focus on a risk-oriented approach rather than a more comprehensive package of care that often results in reduced opportunities for discussion and responding to women’s need for additional information [35, 36, 38].

This study also revealed that information related to preparation for parenthood and postnatal care was rarely provided. A study conducted in England on quality of care showed that women cited three requirements for information from midwives: to help them prepare for parenthood; to enable them to make informed choices; and as a source of reassurance [39]. Postnatal care has been recognised as the area in which women were least likely to be satisfied; the same study revealed that midwives rarely shared aspects of postnatal care that mattered to women, other than support for breastfeeding, which was also demonstrated in this present study. It was suggested that this may be associated with a lack of understanding of women’s priorities among health professionals [35, 36, 39].

Antenatal education is considered essential for influencing the behaviour of women and birth outcome [40–42]. Lack of information provided to the women is often an important factor for not being satisfied with antenatal care [35, 36, 39, 43]. Its comparatively poor compliance as observed in this study raises concerns about the implications for maternal and newborn outcomes. This is especially crucial considering that the main information source used by women during pregnancy to meet their information needs regarding pregnancy, birth and the postpartum period has been the nursing profession. A study found that 70% of the women used “discussion with a midwife” as their source of information, less than half of the women used the internet to access information, and only 2.4% used group information sessions [44].

In essence, the poor compliance to guidelines for antenatal advice might be associated with the risk-oriented approach of care and lack of understanding of women’s priorities. Other studies also found that time might be perceived as a barrier by professionals to improved communication [35]. Training interventions have been shown to improve communication by healthcare professionals; consultations with women by professionals who have undergone communication skills training do not take longer [35].

Overall, a large proportion of the women met the local recommended practices for ultrasound. However, compared to other guidelines from Australia [12], the United Kingdom [11], and the USA [13], the local guidelines for ultrasound were less specific and the recommended initiation was later. These three other guidelines recommend offering early ultrasound in the first trimester. This improves early detection of multiple pregnancies and gestational dating, which may reduce unnecessary postdate pregnancy induction [45]. These guidelines also recommend offering ultrasound at 18–20 weeks to detect structural anomalies [11–13].

The majority of the additional tests were performed on high-risk women, who were expected to require more biological assessment due to their obstetric and/or medical conditions. However, 69% of the low-risk women underwent gestational diabetes testing (MGTT), of whom all were negative. MGTT is the recommended diagnostic/ confirmatory test for women with gestational diabetes mellitus risk factors; women tested positive will be coded as high-risk [10]. The risk factor screening criteria that determines the need for MGTT might need to be reviewed if cost savings are envisaged; especially taking into consideration that the local guidelines also dictated urine sugar screening at every scheduled visit [10]. In comparison, other guidelines do not recommend routine screening for gestational diabetes. Instead, risk factor screening is recommended for the healthy population; women with one or more risk factors will be offered testing for gestational diabetes [11–13].

Full blood picture, iron studies, and haemoglobin electrophoresis were additional investigations for anaemic women whose haemoglobin level was less than 8g% [10]. These tests were not performed at the clinics but were outsourced from the affiliated hospital. Several low-risk women were tested; it was assumed that these women’s haemoglobin had fallen below 8g% during their pregnancy or that they had family history related to haemoglobin disorder that warranted additional screening.

Overall, nearly one-third of the women had urine FEME, which was prescribed when their urine dipstick tested positive for protein or white blood cells or when there was symptom related to urinary tract infection. Malaysia guidelines does not offer routine urine test early in pregnancy to detect asymptomatic bacteriuria. The United Kingdom [11], the USA [13], and Australia [12] recommends to routinely offer urine culture test early in pregnancy to detect asymptomatic bacteriuria. The UK guidelines substantiate its decision based on studies that showed: (i) increased risk between untreated ASB and maternal and foetal outcomes, such as preterm birth and pyelonephritis. (ii) healthcare resource consequences of antenatal ASB screening associated with reduction of maternal and infant morbidity, i.e. future cost saving of treating pyelonephritis and preterm birth as well as the possible resulting lifetime costs of disability associated with preterm birth [11].

### Pregnancy outcomes and risk level

It is known that high-risk women are prone to poorer pregnancy outcomes [27, 30], which was also demonstrated in this present study, emphasising the need for additional attention to this group. The presence of adverse pregnancy outcomes among low-risk women may indicate the importance of other care such as intra-partum care [46, 47]. This may also hint at the need to review the content of ANC provided, especially because a large proportion of low-risk women also had a high level of ANC utilisation.

The updated review comparing reduced visits and standard ANC revealed no clear difference in the pooled number of preterm births and low birth weight in the two groups [48]. Experience from the United States showed that increased ANC utilisation did not reduce LBW [30]. Instead, the authors asserted that risk-oriented ANC may improve the effectiveness of ANC for high-risk women and the efficiency of ANC for low-risk women. Risk assessment is a key component of antenatal care and has demonstrated benefits in promoting improved outcomes [49]. What is crucial is to advocate for vigilant evidence-based risk assessment that upholds the physical, psychologic, and emotional health of the women [49].

Malaysia has been adopting the risk-oriented approach since 1989 to determine care providers, places of ANC and birthing; additional interventions have also been provided according to the care protocols for specific risk conditions of the women. The findings from this study revealed a tendency to focus on the high-risk conditions of the women; and some of the essential ANC content was overlooked. The findings also suggested the opportunity to improve the ANC through reviewing the current guidelines. Upholding the importance of effective ANC that is also responsive to the women’s needs, risk assessment and ANC guidelines must be based on the best available evidence in the scientific literatures and encompass bio-psycho-social perspectives.

### Limitations of the study

As has been discussed elsewhere [31], our study encountered limitations associated with retrospective studies using medical records—evidence of care rendered and quality of the data. Care rendered might not be fully documented. This might result in a slight under-estimation of the proportion of women on whom each of the interventions was performed. Each record was therefore carefully examined for evidence of care rendered but not documented by the provider. The overall documentation quality of the clinics appeared to be satisfactory. Future studies might include an independent validation study to estimate the recording bias.

The ANC content assessment was based on the current national ANC guidelines. In comparison to the evidence-based guidelines from other countries—Australia [12, 50]; United Kingdom [11]; and USA [13]—with better maternal and child health outcomes, in particular Australia and United Kingdom, the Malaysian guidelines might have spaces for improvement. Future studies might also assess ANC content which would include only effective interventions.

## Conclusions

Disproportionate utilisation of ANC among low-risk and high-risk women implies the need to be more attentive to scheduling of care, in particular, intensive utilisation among the low-risk. The risk-oriented approach and lack of understanding of women’s priorities often result in a tendency to focus on the risk factors of high-risk women and specific interventions. Training interventions are recommended to improve communication and to help healthcare professionals understand the priorities of the women. Further studies are required to examine the reason for disproportionate utilisation of ANC according to risk level of pregnancy that would assist in fostering better rational use of services. Studies are also required to assess how delivery of antenatal advice can be improved, reviewing both user and provider perspectives, including the time spent on the risk assessment system that might reduce provider-women interaction.

## Supporting Information

S1 Table Risk Assessment for Pregnant Women. (DOCX)

S2 Table Minimal Requirements for Routine Antenatal Care Content and Compliance Criteria for Scoring. (DOCX)

S3 Table Women Characteristics. (DOCX)
