# Supplementary material for: Antenatal Care Utilisation and Content between Low-Risk and High-Risk Pregnant Women
Source: PLoS One. 2016 Mar 24;11(3):e0152167. doi: 10.1371/journal.pone.0152167 (PMC4807004; doi:10.1371/journal.pone.0152167)
Supplement: S1 Table — (DOCX) [file pone.0152167.s001.docx]

S1 Table.

Supporting Data: Risk assessment for pregnant women

Source: MOH (2010) Perinatal care manual: Section 2 antenatal care (2^nd^ edition).

**CHECKLIST FOR MANAGEMENT OF PREGNANT WOMEN**

|  | RISK FACTORS | Marked (√) if have risk factor | | | | | | |
| --- | --- | --- | --- | --- | --- | --- | --- | --- |
|  | TRIMESTER | 1 | 2 | | 3 | | | Term |
|  | Frequency of risk assessment | 1-12 | 13-22 | 23-27 | 28-31 | 32-35 | 36-40 | >40 |
|  | **CODE RED - IMMEDIATE ADMISSION TO HOSPITAL** | | | | | | | |
| DATE | |  |  |  |  |  |  |  |
| Period of amenorrhea (POA) | |  |  |  |  |  |  |  |
| 1 | Eclampsia |  |  |  |  |  |  |  |
| 2 | Preeclampsia (high blood pressure with urine albumin) or the presence of symptoms or BP >160/110 mmHg |  |  |  |  |  |  |  |
| 3 | Heart disease during pregnancy with signs and symptoms (shortness of breath, palpitations) |  |  |  |  |  |  |  |
| 4 | Shortness of breath when doing light activity (such as sweeping floor, washing dishes) |  |  |  |  |  |  |  |
| 5 | Uncontrolled diabetic mother with presence of urine ketone (≥ 1+) |  |  |  |  |  |  |  |
| 6 | Antepartum haemorrhage (including miscarriage) |  |  |  |  |  |  |  |
| 7 | Abnormal fetal heart rate • FHR ≤ 110 /min on and after 26/52 • FHR> 160 /min after 34/52 (heart rate may be high if premature) |  |  |  |  |  |  |  |
| 8 | Anemia with symptoms at any gestation |  |  |  |  |  |  |  |
| 9 | Premature uterine contractions |  |  |  |  |  |  |  |
| 10 | Liquor without contraction |  |  |  |  |  |  |  |
| 11 | Severe asthmatic attack |  |  |  |  |  |  |  |
| **CODE YELLOW - REFER TO FAMILY MEDICINE SPECIALIST / O&G CLINIC OR NEAREST HOSPITAL (within 48 hours)** | | | | | | | | |
|  | DATE |  |  |  |  |  |  |  |
|  | POA |  |  |  |  |  |  |  |
| 1 | HIV positive mother |  |  |  |  |  |  |  |
| 2 | Hepatitis-B positive mother |  |  |  |  |  |  |  |
| 3 | High blood pressure >140/90 - <160/110 mmHg with negative urine albumin |  |  |  |  |  |  |  |
| 4 | Diabetic mother |  |  |  |  |  |  |  |
| 5 | Reduced fetal movement at ≥ 32 weeks |  |  |  |  |  |  |  |
| 6 | Pregnancy exceeding 7 days from the DD |  |  |  |  |  |  |  |
| 7 | Mother with a medical problem that requires treatment with hospital |  |  |  |  |  |  |  |
|  |  |  |  |  |  |  |  |  |
| 8. | Mother involved in Medico-legal issues |  |  |  |  |  |  |  |
| 9 | Single mother and teenage mother |  |  |  |  |  |  |  |
| 10 | Haemoglobin <9.5gm% |  |  |  |  |  |  |  |

**CODE GREEN - REFER TO THE MEDICAL OFFICER (MO/ FMS)**

|  | RISK FACTORS | Marked (√) if have risk factor | | | | |
| --- | --- | --- | --- | --- | --- | --- |
|  | TRIMESTER | 1 | 2 | | 3 | |
| TARIKH | | 1-12 | 13-20 | 21-28 | 29-32 | 33-36 |
| POA | |  |  |  |  |  |
| 1 | *Rh negative |  |  |  |  |  |
| 2 | * Mother's weight before pregnancy or at booking <45kg |  |  |  |  |  |
| 3 | * Current medical problems (including psikiarik and physical disability) |  |  |  |  |  |
| 4 | * Past gynecological surgery |  |  |  |  |  |
| 5 | * Drugs addiction / smoking |  |  |  |  |  |
| 6 | * LNMP uncertain |  |  |  |  |  |
| 7 | * 3 consecutive miscarriages |  |  |  |  |  |
| 8 | Past obstetric history: |  |  |  |  |  |
|  | i) Caesarean section |  |  |  |  |  |
|  | ii) past history of pregnancy induced hypertension / Eclampsia / Diabetes |  |  |  |  |  |
|  | iii) Perinatal Mortality |  |  |  |  |  |
|  | iv) History of infants weighing less than 2.5kg or more than 4kg |  |  |  |  |  |
|  | v) 3rd degree perineum tear |  |  |  |  |  |
|  | vi) Retained placenta |  |  |  |  |  |
|  | vii) Bleeding after childbirth |  |  |  |  |  |
|  | viii) Instrumental birth |  |  |  |  |  |
|  | ix) long labour pain |  |  |  |  |  |
| 9 | Multiple pregnancy |  |  |  |  |  |
| 10 | High blood pressure (140/90 mmHg) without urinary albumin |  |  |  |  |  |
| 11 | Hamoglobin less than 11g% |  |  |  |  |  |
| 12 | Glucose detected in urine 2 times |  |  |  |  |  |
| 13 | Urine albumin ≥ 1+ |  |  |  |  |  |
| 14 | Sudden weight gain of more than 2 kg in a week |  |  |  |  |  |
| 15 | Weigh more than 80 kg at "booking" |  |  |  |  |  |
| 16 | Uterine height (SFH) smaller or larger from the estimated period of gestation |  |  |  |  |  |
| 17 | Oblique/ transverse with no sign of labor at 36 weeks of pregnancy |  |  |  |  |  |
| 18 | (Head not engaged) at term (37 weeks) for primigravida |  |  |  |  |  |

* One-time assessment only

Note: The mother must be examined by the Medical Officer within 2 weeks from the date of booking

**CODE WHITE (HOSPITAL DELIVERY)**

| **RISK FACTORS** | | **RISK** |
| --- | --- | --- |
| DATE | |  |
| POA | |  |
| 1 | Primigravida |  |
| 2 | Mother younger than 18 or older than 40 years |  |
| 3 | Gravida 6 and above |  |
| 4 | Spacing of less than 2 years or more than 5 years |  |
| 5 | Mother with specific problems: i) height of less than 145 cm |  |

**CODE WHITE (ALLOWED TO DELIVERY AT HOME / ALTERNATIVE BIRTHING CENTER)**

If fulfills the following conditions

| **RISK FACTORS** | | **RISK** |
| --- | --- | --- |
| DATE | |  |
| POA | |  |
| 1 | Gravida 2-5 |  |
| 2 | No previous obstetrics problem |  |
| 3 | No previous medical problem |  |
| 4 | No complications during pregnancy |  |
| 5 | Appropriate home environment |  |
| 6 | Height more than 145cm |  |
| 7 | Mother more than 18 years old and less than 40 years |  |
| 8 | Married and have family support |  |
| 9 | POA >37 weeks or <41 weeks |  |
| 10 | Estimated baby weight >2 kg and <3.5 kg |  |
