# Supplementary material for: Antenatal Care Utilisation and Content between Low-Risk and High-Risk Pregnant Women
Source: PLoS One. 2016 Mar 24;11(3):e0152167. doi: 10.1371/journal.pone.0152167 (PMC4807004; doi:10.1371/journal.pone.0152167)
Supplement: S2 Table — (DOCX) [file pone.0152167.s002.docx]

S2 Table.

Supporting Data: Recommended Routine Antenatal Care Content and Compliance Criteria for Scoring

| # | **Routine care listed in MOH Guidelines** | **Compliance criteria for scoring** | **score** |
| --- | --- | --- | --- |
| **I)** | **PHYSICAL EXAMINATION (PE)** |  |  |
| 1 | oral hygiene (or referral for oral health services) | at least once | 1 |
| 2 | general condition - pallor, cyanosis, varicose veins, etc. | at booking and subsequent visits | 1 |
| 3 | cardiovascular system | at least 2 times | 1 |
| 4 | respiratory | at least 2 times | 1 |
| 5 | thyroid | at least 2 times | 1 |
| 6 | abdomen - previous scar/ other masses | at booking and during RME | 1 |
| 7 | height | indicated done (value found) | 1 |
| 8 | weight | as per recommended visits - 10 times for primigravida, 7 times for multigravida at POG 40 weeks; adjusted for POG at birth.  If total visits is less than 10 or 7, evidence of being done at each visit is accepted. | 1 |
| 9 | blood pressure | as per recommended visits - 10 times for primigravida, 7 times for multigravida at POG 40 weeks; adjusted for POG at birth.  If total visits is less than 10 or 7, evidence of being done at each visit is accepted. | 1 |
| 10 | breast | at least once | 1 |
| 11 | symphysis-fundal height | from 22 weeks onwards (include those done 1 week earlier) - 8 times for primigravida, 5 times for multigravida at POG 40 weeks; adjusted for POG at birth.  If total visits is lesser than this number, evidence of being done at each visit is accepted. | 1 |
| 12 | foetal lie and presentation | from 32 weeks onwards (include those done 1 week earlier) - 6 times for primigravida, 4 times for multigravida at POG 40 weeks; adjusted for POG at birth.  If total visits is lesser than this number, evidence of being done at each visit is accepted. | 1 |
| 13 | foetal heart auscultation | from 24 weeks onwards (include those done 1 week earlier) - 8 times for primigravida, 5 times for multigravida at POG 40 weeks; adjusted for POG at birth.  If total visits is lesser than this number, evidence of being done at each visit is accepted. | 1 |
| 14 | oedema | as per recommended visits - 10 times for primigravida, 7 times for multigravida at POG 40 weeks; adjusted for POG at birth.  If total visits is less than 10 or 7, evidence of being done at each visit is accepted. | 1 |
|  |  | **Physical Examination (PE)** | **14** |
| **II)** | **HEALTH SCREENING (HS)** |  |  |
| 1 | urine protein | as per recommended visits - 10 times for primigravida, 7 times for multigravida at POG 40 weeks; adjusted for POG at birth.  If total visits is less than 10 or 7, evidence of being done at each visit is accepted. | 1 |
| 2 | urine sugar | as per recommended visits - 10 times for primigravida, 7 times for multigravida at POG 40 weeks; adjusted for POG at birth.  If total visits is less than 10 or 7, evidence of being done at each visit is accepted. | 1 |
| 3 | haemoglobin or full blood count | 50% of recommended visits - 5 times for primigravida, 4 times for multigravida at POG 40 weeks; adjusted for POG at birth.  If total visits less than 5 or 4, evidence of being done at each visit is accepted. | 1 |
| 4 | ABO blood grouping | once | 1 |
| 5 | Rhesus factor blood test | once | 1 |
| 6 | VDRL | once | 1 |
| 7 | HIV | once | 1 |
| 8 | Ultrasound, abdominal | at least two times, including ultrasound done by other provider; acceptable if POG of 1st visit was >24 weeks, and only 1 ultrasound done after 24 weeks. | 1 |
| 10 | Hepatitis B | once | 1 |
|  |  | **Health Screening (HS)** | **9** |
| **III)** | **CASE MANAGEMENT (CM)** |  |  |
| 1 | routine medical examination (RME) by doctor - 1^st^ RME | RME1 at booking or by POG 24 weeks (plus 1 week acceptable). | 1 |
| 2 | routine medical examination (RME) by doctor – 2^nd^ RME | RME2 at 31-36 weeks (± 1 week is acceptable); adjusted for POG at birth. | 1 |
| 3 | risk assessment | at 1-12 weeks; ±1 week; adjusted for initiation and POG at birth. | 1 |
|  |  | at 13-20 weeks; ±1 week; adjusted for initiation and POG at birth. |  |
|  |  | at 21-28 weeks; ±1 week; adjusted for initiation and POG at birth. |  |
|  |  | at 29-32 weeks; ±1 week; adjusted for initiation and POG at birth. |  |
|  |  | at 33-36 weeks or 33 weeks onwards; ±1 week; adjusted for initiation and POG at birth. |  |
| 4 | colour tagging of risk | appropriate risk tagging, compared against known risk factors/ past history | 1 |
| 5 | ultrasound performed routine before or at 24 weeks of pregnancy | at least once before or at POG 24 weeks (plus 1 week is acceptable), including U/S done by other provider;  Acceptable if 1st visit >24 weeks in which no previous U/S done by other provider and HC did U/S at 1st visit or later;  Acceptable if 1st visit >24 weeks in which U/S done by other provider previously regardless of POG.  Not acceptable if 1st visit >24 weeks and no U/S done throughout. | 1 |
| 6 | immunisation - anti-tetanus vaccination (in dose) | two doses for primigravida, one booster dose for multigravida (if completed 2 doses at Gravidity 1). | 1 |
| 7 | haematinic supplement (include folic acid or multivitamins supplements) | must be prescribed at booking to avoid missed-opportunity of taking folic acid before POG 12 weeks. | 1 |
|  |  | **Case Management (CM)** | **7** |
| **IV)** | **HEALTH EDUCATION (HE)** (based on listing in ANC booklet) |  |  |
| 1 | nutritional/dietary advice - antenatal | at least once | 1 |
| 2 | nutritional/dietary advice - postnatal/ breastfeeding | at least once | 1 |
| 3 | recommendations for family planning/ contraception | at least once | 1 |
| 4 | preparation for birth | at least once | 1 |
| 5 | birth process (S&S and related advice) | at least once | 1 |
| 6 | common discomfort during pregnancy and solutions | at least once | 1 |
| 7 | recommendations for breastfeeding | at least once | 1 |
| 8 | common disorders in pregnancy | at least 2 out of 5 listed topics (pregnancy induced hypertension, preeclampsia/ impending eclampsia, gestational diabetes mellitus, anaemia, bleeding) | 1 |
| 9 | early booking | at least once | 1 |
| 10 | foetal development | at least once | 1 |
| 11 | exercise antenatal/ postnatal | at least once | 1 |
| 12 | newborn care, baby bathing | at least once | 1 |
| 13 | jaundice baby care | at least once | 1 |
| 14 | postnatal care | at least once | 1 |
|  |  | **Health Education (HE)** | **14** |
|  |  | **MAXIMUM COMPLIANCE SCORE** | **44** |

Source: Ministry of Health, 2010. Perinatal care manual: Section 2 antenatal care (2^nd^ edition).
