# Supplementary material for: Antenatal Care Utilisation and Content between Low-Risk and High-Risk Pregnant Women
Source: PLoS One. 2016 Mar 24;11(3):e0152167. doi: 10.1371/journal.pone.0152167 (PMC4807004; doi:10.1371/journal.pone.0152167)
Supplement: S3 Table — (DOCX) [file pone.0152167.s003.docx]

S3 Table.

Supporting Data: Characteristics of the women by risk level of pregnancy (n=522)

| **Characteristics** |  | **Unit** | **All women**  **(n=522)** | **Low-risk**  **(n=375)** | **High-risk**  **(n=147)** |
| --- | --- | --- | --- | --- | --- |
| **Maternal age at first visit** | <=19 | n (%) | 11 (2.1) | 6 (1.6) | 5 (3.4) |
|  | 20-34 | n (%) | 439 (84.1) | 327 (87.2) | 112 (76.2) |
|  | >=35 | n (%) | 72 (13.8) | 42 (11.2) | 30 (20.4) |
|  | Mean (SD) |  | 28.7 (5.0) | 28.3 (4.7) | 29.6 (5.5) |
| **Ethnicity** | Malay | n (%) | 396 (75.9) | 297 (79.2) | 99 (67.3) |
|  | Chinese | n (%) | 67 (12.8) | 41 (10.9) | 26 (17.7) |
|  | Indian | n (%) | 44 (8.4) | 27 (7.2) | 17 (11.6) |
|  | Indigenous people | n (%) | 15 (2.9) | 10 (2.7) | 5 (3.4) |
| **Maternal education** | Primary or no education | n (%) | 23 (4.4) | 12 (3.2) | 11 (7.5) |
|  | Secondary | n (%) | 294 (56.3) | 209 (56.7) | 85 (57.8) |
|  | Tertiary | n (%) | 193 (37.0) | 148 (39.5) | 45 (30.6) |
|  | unknown | n (%) | 12 (2.3) | 6 (1.6) | 6 (4.1) |
| **Maternal occupation** | Legislators, senior officials and managers; professionals and associate professionals | n (%) | 131 (25.1) | 99 (26.4) | 32 (21.8) |
|  | Non managerial and non-professional workers  (incl. clerical support, service/sales, craft and related trades, plant/machine, elementary workers) | n (%) | 188 (36.0) | 137 (36.5) | 51 (34.7) |
|  | Non formal employment  (housewives, students, unemployed) | n (%) | 199 (38.1) | 135 (36.0) | 64 (43.5) |
|  | unknown | n (%) | 4 (0.8) | 4 (1.1) | 0 (0.0) |
| **Parity** | Nulliparity | n (%) | 195 (37.4) | 142 (37.9) | 53 (36.1) |
|  | Multiparity | n (%) | 327 (62.6) | 233 (62.1) | 94 (63.9) |
|  | Mean (SD) |  | 1.2 (1.3) | 1.1 (1.2) | 1.4 (1.5) |
| **Risk code** | White | n (%) | 160 (30.7) | 160 (42.7) | NA |
|  | Green | n (%) | 215 (41.2) | 215 (57.3) | NA |
|  | Yellow | n (%) | 140 (26.8) | NA | 140 (95.2) |
|  | Red | n (%) | 7 (1.3) | NA | 7 (4.8) |
| **Clinic type (by planned daily workload)** | <150 | n (%) | 98 (18.8) | 69 (18.4) | 29 (19.7) |
|  | 150-300 | n (%) | 247 (47.3) | 182 (48.5) | 65 (44.2) |
|  | 301-500 | n (%) | 177 (33.9) | 124 (33.1) | 53 (36.1) |
| **Adequacy of initiation of care (modified APNCU Index)** | Inadequate | n (%) | 96 (18.4) | 66 (17.6) | 30 (20.4) |
|  | Adequate | n (%) | 426 (81.6) | 309 (82.4) | 117 (79.6) |
| **Adequacy of observed-to-expected visits ratio** | Inadequate | n (%) | 27 (5.2) | 19 (5.1) | 8 (5.4) |
|  | Adequate | n (%) | 495 (94.8) | 356 (94.9) | 139 (94.6) |
| **Adequacy of ANC utilisation** | Inadequate | n (%) | 107 (20.5) | 75 (20.0) | 32 (21.8) |
|  | Adequate | n (%) | 85 (16.3) | 79 (21.1) | 6 (4.1) |
|  | Adequate-plus | n (%) | 330 (63.2) | 221 (58.9) | 109 (74.1) |
| **Adequacy of ANC content** | Inadequate (<80%) | n (%) | 270 (51.7) | 181 (48.3) | 89 (60.5) |
|  | Adequate (>80%) | n (%) | 252 (48.3) | 194 (51.7) | 58 (39.5) |
